# Tonic neuron position and network topology determine synchronization dynamics

**DOI:** 10.1007/s11571-026-10585-2

**Published:** 2026-11-02

**Authors:** Agustín Farrera Megchun, Pablo Padilla, Jesús Espinal-Enríquez, Gerardo J. Escalera Santos, Roberto Bernal-Jaquez

**Affiliations:** 1 Posgrado en Ciencias Naturales e Ingeniería, Universidad Autónoma Metropolitana-Cuajimalpa, Ciudad de México, 05348 México; 2 Instituto de Investigaciones en Matemáticas Aplicadas y en Sistemas, Departamento de Matemáticas y Mecánica, Universidad Nacional Autónoma de México, Ciudad de México, 04510 México; 3 Computational Genomics Division, National Institute of Genomic Medicine, Mexico City, 14610 Mexico; 4 Facultad de Ciencias en Física y Matemáticas, Universidad Autónoma de Chiapas, Tuxtla Gutiérrez, 29050 Chiapas México; 5 Departamento de Matemáticas Aplicadas y Sistemas, Universidad Autónoma Metropolitana-Cuajimalpa, Ciudad de México, 05348 México

**Keywords:** Network topology, Synchronization dynamics, Firing patterns, Tonic and bursting neurons, Huber–Braun model, Partial firing death

## Abstract

Understanding how collective neuronal rhythms emerge from the interplay between network structure and single-neuron dynamics remains a fundamental challenge in neurodynamics. In this work, we show that in small heterogeneous networks, the network topology and the position of a single tonic neuron govern synchronization dynamics. We study minimal four-neuron Huber–Braun motifs composed of one tonic neuron and three bursting neurons coupled via electrical synapses. By systematically varying the tonic neuron’s centrality—quantified through degree and a quasi-Laplacian measure—we identify two critical coupling thresholds: the critical coupling for global phase synchronization and the coupling at which the network returns to synchronized periodic bursting. Remarkably, centrality determines the nature of the synchronized attractor: motifs in which the tonic neuron has low centrality synchronize into tonic spiking, whereas highly central tonic neurons drive the network toward synchronized bursting. Moreover, the networks exhibit nontrivial collective behaviors, including large-ISI “stairway-to-heaven” structures, partial firing death characterized by the coexistence of spiking and subthreshold neurons, and remote synchronization in the star configuration with the tonic neuron at the hub. While the present study focuses on the Huber–Braun model in four-neuron motifs, excitability and electrical coupling are features shared by many conductance-based neuronal models. This motivates the hypothesis that analogous structure–dynamics relationships may also arise in other neuronal models and small network motifs, highlighting the potential role of local structural control in shaping collective neural rhythms.

## Introduction

Synchronization is a collective behavior observed in a variety of natural systems, where individual units interact to coordinate their dynamics (Pikovsky et al. 2001; Boccaletti et al. 2018; Arenas et al. 2008; Boccaletti 2008). In the brain, neuronal synchronization is essential for functions such as memory, vision, and information processing (Roelfsema et al. 1997; Fell and Axmacher 2011; Singer 1993). However, excessive synchronization is associated with neurological disorders. For instance, seizures in epilepsy involve highly synchronized neural activity (Jiruska et al. 2013), and patients with Parkinson’s disease and tremor exhibit abnormal in-phase synchrony in subthalamic nucleus neurons (Levy et al. 2000). Understanding the mechanisms that govern synchronization in neuronal networks is therefore a central problem in theoretical neuroscience.

Neuronal firing displays diverse patterns, including tonic spiking, chaotic spiking, and both periodic and chaotic bursting. Mixed populations of tonic-spiking and bursting neurons are common in the cortex and thalamocortical systems (Sherman 2001). Tonic spiking consists of regular, high-frequency action potentials, whereas bursting comprises episodes of rapid spiking separated by quiescent intervals. These patterns occur across the nervous system in distinct functional contexts, such as sensory systems, thalamocortical information relay, regulation of vigilance, and during sleep and wakefulness (Contreras et al. 1992; Sherman and Guillery 1428; Weyand et al. 2001). Moreover, these dynamics—especially when highly synchronized—are implicated in pathological conditions such as epileptic seizures and Parkinson’s disease (Hofer et al. 2022; Tai 2021). Consequently, transitions between tonic and bursting firing patterns have become a major focus of both theoretical and experimental research (Li et al. 2023; Zhou et al. 2021; Gu 2013; Gu et al. 2014).

The study of small networks, such as network motifs, reveals that they act as fundamental building blocks of brain organization (Milo et al. 2002). Motif analysis shows that specific connectivity patterns are overrepresented and play a crucial role in shaping network stability, information processing, and functional diversity in the brain (Sporns and Kötter 2004; Battiston et al. 2017; Song et al. 2005). For example, focal seizures often begin in a small, localized region of the brain and are believed to be initiated by small pacemaker-like neuronal populations (Meeren et al. 2005). Indeed, several studies indicate that seizures frequently originate within dysfunctional microcircuits (see review (Paz and Huguenard 2015)). Supporting this view, single-unit recordings in the human neocortex have shown that seizure activity is accompanied by a pronounced coexistence of firing regimes within small cortical patches. In particular, Truccolo et al. (2011) reported that during epileptic seizures some neurons exhibit high-frequency spiking while others strongly reduce or transiently suppress their activity, even within the same microdomain. These observations motivate the study of minimal neuronal motifs in which the functional role of individual neurons can be systematically controlled and analyzed.

In this study, we use the Huber–Braun (HB) model to analyze the behavior of coupled neurons in small networks with different topologies. This biologically inspired, conductance-based model was originally proposed to reproduce spike trains observed in cold receptors (Braun et al. 1998). In recent years, owing to its rich dynamics and biological grounding, the HB model has been applied to various problems, including complex behaviors driven by perturbations (Megchun et al. 2025; Boaretto et al. 2025), synchronization dynamics (Boaretto et al. 2021; Budzinski et al. 2019; Prado et al. 2014; Farrera-Megchun et al. 2025), complex wave propagation (Rajagopal et al. 2023; Wang et al. 2007), sleep–wake cycle simulations (Hopkins et al. 2018; Qiu et al. 2021), and seizure modeling (Follmann et al. 2024). These studies demonstrate that the HB model provides a flexible and robust framework for investigating synchronization, firing-pattern transitions, and collective dynamics with biological realism. In particular, tonic-to-bursting transitions and synchronization properties have been extensively examined within this model (Shaffer et al. 2016, 2017; Follmann et al. 2018; Postnova et al. 2007).

More recently, neuronal arrangement was shown to influence synchronization and firing-pattern transitions within a fixed ring topology (Farrera-Megchun et al. 2024), whereas the effect of pacemaker position was examined in selected directed HB networks (Farrera-Megchun et al. 2025). Despite these advances, it remains unclear whether the structural position of a single dynamically distinct neuron can determine not only when synchronization occurs but also which collective firing regime emerges. This question differs from that addressed in our previous studies: here, the tonic neuron is not an imposed pacemaker but a dynamical unit whose activity can be modified through bidirectional electrical coupling with the bursting neurons. We therefore analyze all eleven connected four-node configurations composed of one tonic neuron and three bursting neurons to determine how topology and tonic-neuron position organize synchronization and firing-pattern transitions.

Our results show that topology affects the nature of the collective dynamics, rather than merely shifting the synchronization threshold. At the onset of synchronization, configurations in which the tonic neuron has degree one synchronize in tonic-spiking dynamics, whereas those in which it has degree three synchronize in bursting dynamics. At stronger coupling, all configurations eventually converge to synchronized periodic bursting through topology-dependent firing-pattern transitions. The critical couplings decrease with the quasi-Laplacian centrality of the tonic neuron and are summarized by empirical power-law fits. Moreover, the motifs exhibit partial firing death, large-ISI “stairway-to-heaven” structures, and remote synchronization in the star configuration with the tonic neuron at the hub. These behaviors, which were not analyzed in our previous studies, demonstrate that the structural role of the tonic neuron selects among qualitatively different collective dynamical states.

The manuscript is organized as follows. Section “Model and methods” introduces the model and methodology. Section “Numerical results” presents the results in two parts: first, the effects on the critical coupling; second, the effects on firing-pattern transitions. Finally, Sect. “Discussion and conclusion” provides the discussion and conclusions.

## Model and methods

### The Huber-Braun model

The HB model is based on the Hodgkin-Huxley framework and consists of four differential equations describing both physical and physiological properties of a neuron (Braun et al. 1998). The membrane potential evolves according to

1 $$\begin{aligned} C_{m}\frac{dV}{dt}=-I_{l}-I_{d}-I_{r}-I_{sd}-I_{sr}, \end{aligned}$$

where $C_{m}$ is the membrane capacitance and $I_{l}=g_{l}(V-V_{l})$ is the leakage current. $I_{d}$ and $I_{r}$ are fast currents corresponding to sodium (Na$^{+}$) and potassium (K$^{+}$) channels, respectively, and are responsible for spike generation. $I_{sd}$ and $I_{sr}$ are the slow currents for subthreshold oscillations. Each current is expressed as

2 $$\begin{aligned} I_{x}=\rho (T) g_{x} a_{x}(V-V_{x}), \ \ \ x=d,r,sd,sr, \end{aligned}$$

here *ρ * is the temperature-dependent scaling factor for the ionic currents, $g_x$ is the maximal conductance, $V_x$ is the equilibrium potential and $a_{x}$ represents the activation variable. Since the sodium current $I_d$ changes much faster than other ionic currents, i.e., the depolarization current is activated instantly, we consider that:

3 $$\begin{aligned} a_{d}=a_{d_{\infty }}. \end{aligned}$$

$I_{r}$ and $I_{sd}$ are activated with characteristic time constants $\tau _{r}$ and $\tau _{sd}$, respectively:

4 $$\begin{aligned} \frac{da_{r}}{dt}=\frac{\phi (T)(a_{r_{\infty }}-a_{r})}{\tau _{r}}, \end{aligned}$$

5 $$\begin{aligned} \frac{da_{sd}}{dt}=\frac{\phi (T)(a_{sd_{\infty }}-a_{sd})}{\tau _{sd}}, \end{aligned}$$

where the steady-state activation variables are represented by sigmoidal functions:

6 $$\begin{aligned} a_{x_{\infty }}=\frac{1}{1+\text {exp}(-s_{x}(V-V_{0_{x}}))}, \ \ \ x=r,sd,d, \end{aligned}$$

with $s_{x}$ the activation slope and $V_{0_{x}}$ the half-activation potential. The slow repolarization current $I_{sr}$ is activated and relaxed in a constant time $\tau _{sr}$:

7 $$\begin{aligned} \frac{da_{sr}}{dt}=\frac{\phi (T)(-\eta I_{sd}-\theta a_{sr})}{\tau _{sr}}, \end{aligned}$$

*η * takes into account the increase in calcium ions associated with $I_{sd}$ and *θ * is a factor for the relaxation. Temperature dependent factors are defined as:

8 $$\begin{aligned} \rho =1.3^{\frac{T-T_0}{10}}, \ \phi =3.0^{\frac{T-T_0}{10}}, \end{aligned}$$

where 1.3 and 3.0 represent the $Q_{10}$ temperature coefficients for the maximum conductances and the time constants of current activation, respectively (Hille 1992; Hodgkin et al. 1952). These values reflect the sensitivity of ion channels to temperature.

In numerical simulations the parameter values are $g_{l} = 0.1$, $g_{d}=1.5$, $g_{r}=2$, $g_{sd}=0.25$, $V_{sd}=V_{d}=50$, $V_{sr}=V_r=-90$, $V_l=-60$, $\tau _{r}=2$, $\tau _{sd}= 10$, $\tau _{sr}= 20$, $s_{d}=s_r = 0.25$, $s_{sd} = 0.09$, $V_{0d} = V_{0r} = -25$, $V_{0sd} = -40$, *η = 0.012*, *θ =0.17*, $T_{0}=25$, *T=25*, and $C_{m}$ = 1. The conductances in $\text {mS}/\text {cm}^2$, the equilibrium potentials in $\text {mV}$, the time constants in $\text {ms}$, the slopes of steady state activation in $\text {mV}^{-1}$, the *η * factor in $\text {cm}^{2}/\mu \text {A}$, and the temperatures in °C.

### Neural connectivity configuration

We consider network motifs composed of four HB neurons coupled via electrical synapses: three bursting neurons and one tonic neuron. The membrane potential dynamics of each neuron is described by

9 $$\begin{aligned} C_{m}\frac{dV_{i}}{dt}=-I_{i,l}-I_{i,d}-I_{i,r}-I_{i,sd}-I_{i,sr}-g_{c}\sum _{j}A_{ij}(V_{i}-V_{j}), \end{aligned}$$

where $V_{i}$ and $V_{j}$ are the membrane voltages of *i*-th and *j*-th neurons, respectively. The last term represents the electrical coupling with constant strength $g_{c}$. We assumed that all connections between neurons are bidirectional. The connectivity of the network is defined by an adjacency matrix $A_{ij}$, where the elements $A_{ij}= A_{ji}=1$ if the neurons *i* and *j* are connected, $A_{ij}=0$, otherwise, and we consider no self-connections, which means $A_{ii}=0$.

In our numerical simulations, the fourth-order Runge-Kutta method is used to solve the differential equations with *dt=0.01*ms for a randomly distributed set of initial conditions in the intervals $V_{i} \in [-65,0]$ mV and $a_{x_{i}} \in [0,1]$, where *x* represents *r*, *sd*, and *sr*.

### Measuring synchronization

Neuronal synchronization is quantified using the Kuramoto order parameter, *R*, which characterizes global phase synchrony (Kuramoto 1984). It is defined as:

10 $$\begin{aligned} R(t)=\Big | \frac{1}{N}\sum _{j} e^{i\phi _{j}(t)} \Big | , \end{aligned}$$

where $\phi _{j}(t)$ is the phase associated with each neuron’s membrane potential

11 $$\begin{aligned} \phi _{j}(t)=2\pi \frac{t-t_{k,j}}{t_{k+1,j}-t_{k,j}}+2\pi k_{j}, \ \ \ t_{k,j}\le t < t_{k+1,j}, \end{aligned}$$

where $t_{k,j}$ is the time of the *k*-th spikes of the *j*-th neuron and $t_{k+1,j}$ is the time of the following *(k + 1)*-th spikes. The time-averaged order parameter is then:

12 $$\begin{aligned} \langle R \rangle =\frac{1}{t_{f}-t_{i}}\sum _{t=t_i}^{t_f} R(t), \end{aligned}$$

where $t_{i}$ and $t_{f}$ are the start and end times for calculating the order parameter *R*(*t*), which takes values between 0 (for an unsynchronized state) and 1 (for a phase-synchronized state). Because the phase definition requires consecutive spike events, *R*(*t*) was evaluated only at times for which the phases of all four neurons were simultaneously defined. If any neuron was nonspiking or fewer than two spike events were detected, $\langle R\rangle$ was considered undefined. In our simulations, $\langle R \rangle$ is calculated from $t_{i}= 270$s to $t_{f}= 300$s. The coupling strength $g_{c}$ is gradually increased ($\delta g_{c}=0.0025$) using forward continuation: the final state at each $g_{c}$ step is used as the initial condition for the next. All values of $g_{c}$ are in millisiemens per square centimeter, $mS/cm^2$. To investigate the effects of different network topologies on synchronization, we determined the critical coupling strength, $g_{c}^{*}$, for each network. This quantity represents the minimum coupling at which all four neurons are spiking and the system reaches a globally synchronized state. Specifically, $g_c^*$ is defined as the minimum value of $g_c$ for which $\langle R\rangle \ge 0.98$.

To characterize remote synchronization in the star network, we additionally computed the pairwise phase coherence as defined in Bergner et al. (2012):

13 $$\begin{aligned} r_{nm}= \left| \left\langle \exp \left\{ i[\phi _n(t)-\phi _m(t)]\right\} \right\rangle _t \right| , \end{aligned}$$

where the phases are defined from consecutive spike times according to Eq. 11. Denoting the central neuron by *h* and the three peripheral neurons by $p_1,p_2,p_3$, we define

14 $$\begin{aligned} r_{\textrm{direct}} = \frac{1}{3}\sum _{j=1}^{3}r_{hp_j}, \qquad r_{\textrm{indirect}} = \frac{1}{3}\sum _{j<k}r_{p_jp_k}. \end{aligned}$$

The first quantity measures the mean coherence between the hub and the peripheral neurons, whereas the second measures the mean coherence among the three peripheral neurons, which have no direct connections. A time sample was included only when the spike-defined phases of all four neurons were simultaneously defined.

For the classification of firing-death states, spike events were identified as local maxima of the membrane potential exceeding $V_{\textrm{th}}=-20$ mV. A neuron was classified as nonspiking (subthreshold) when no spike event was detected during the stationary interval *t∈ [270,300]* s. Partial firing death was defined as the coexistence of spiking and nonspiking neurons, that is, when at least one but fewer than four neurons were classified as nonspiking. In contrast, complete firing death was defined as the absence of detected spikes in all four neurons during the same interval.

The power-law model, $y=AL_Q^B$, and the exponential model, $y=A\exp (BL_Q)$, were fitted to the 11 mean critical-coupling values by ordinary least squares after logarithmic transformation. Goodness of fit was evaluated in the transformed space using $R^{2}=1-\sum _i(z_i-\widehat{z}_i)^2/\sum _i(z_i-\overline{z})^2$, where $z_i=\ln y_i$, $\widehat{z}_i$ is the value predicted by the fitted model, and $\overline{z}$ is the sample mean of the transformed observations. The 95% confidence intervals for the fitted parameters were calculated using Student’s *t* distribution with *n-2* degrees of freedom. Topologically distinct configurations sharing the same $L_Q$ were retained as separate observations.

## Numerical results

**Fig. 1:**
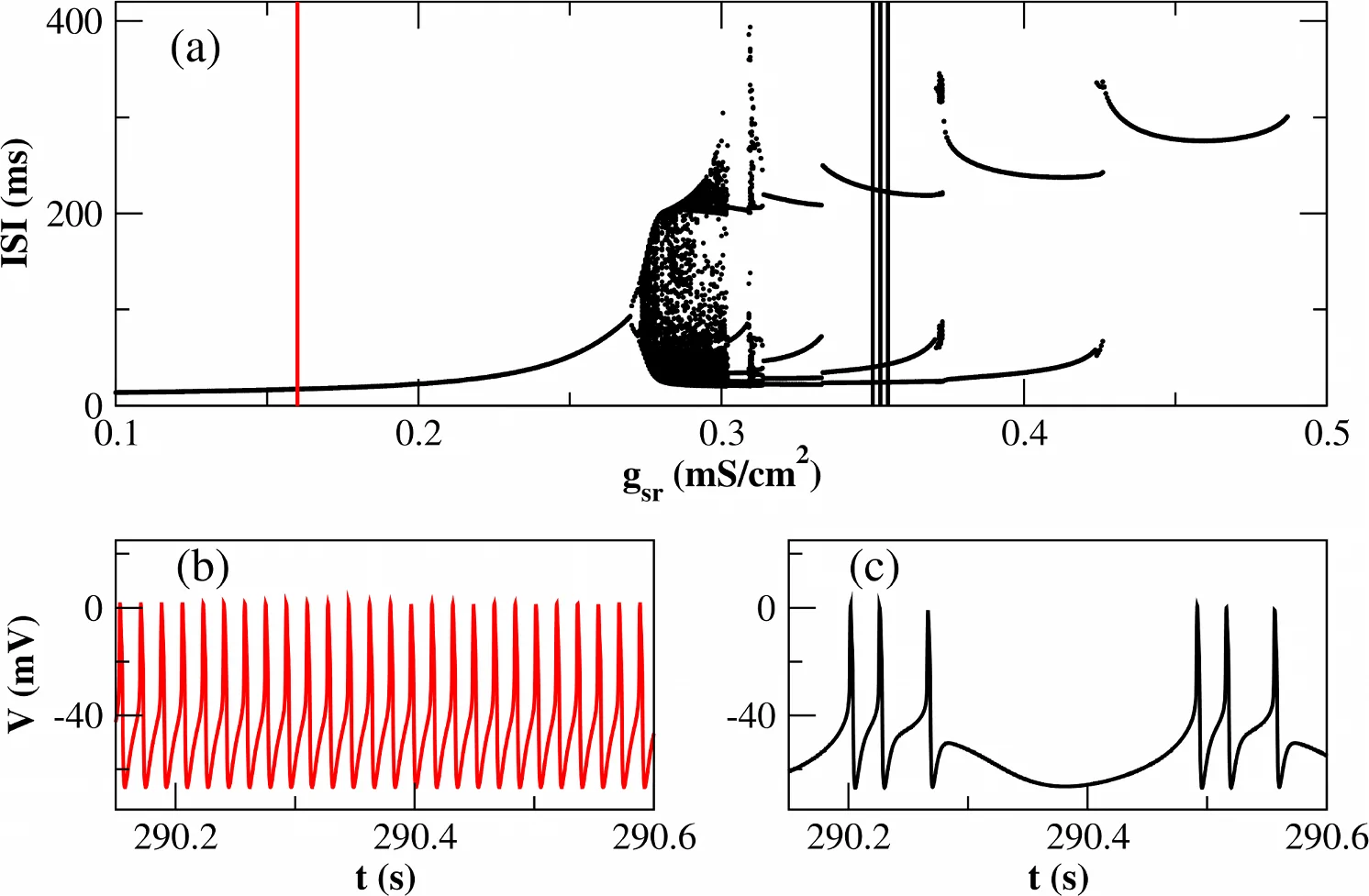
**a** The ISI bifurcation of an uncoupled neuron as a function of the maximal conductance $g_{sr}$. Time series from the membrane potentials for two different values of $g_{sr}$: **b** 0.16 and **c** 0.35. The red line indicates the value $g_{sr}=0.16$ for tonic spiking, whereas the three black lines indicate the values $g_{sr} = \{0.35, 0.3525, 0.355\}$

In this section, we present the results, focusing on synchronization and firing-pattern transitions. To obtain tonic spiking dynamics, we set the parameter $g_{sr}=0.16$. For bursting dynamics (three spikes per burst), we use $g_{sr}$ values of 0.35, 0.3525, and 0.355 (see Fig. 1).

### Effects on the critical coupling

**Fig. 2:**
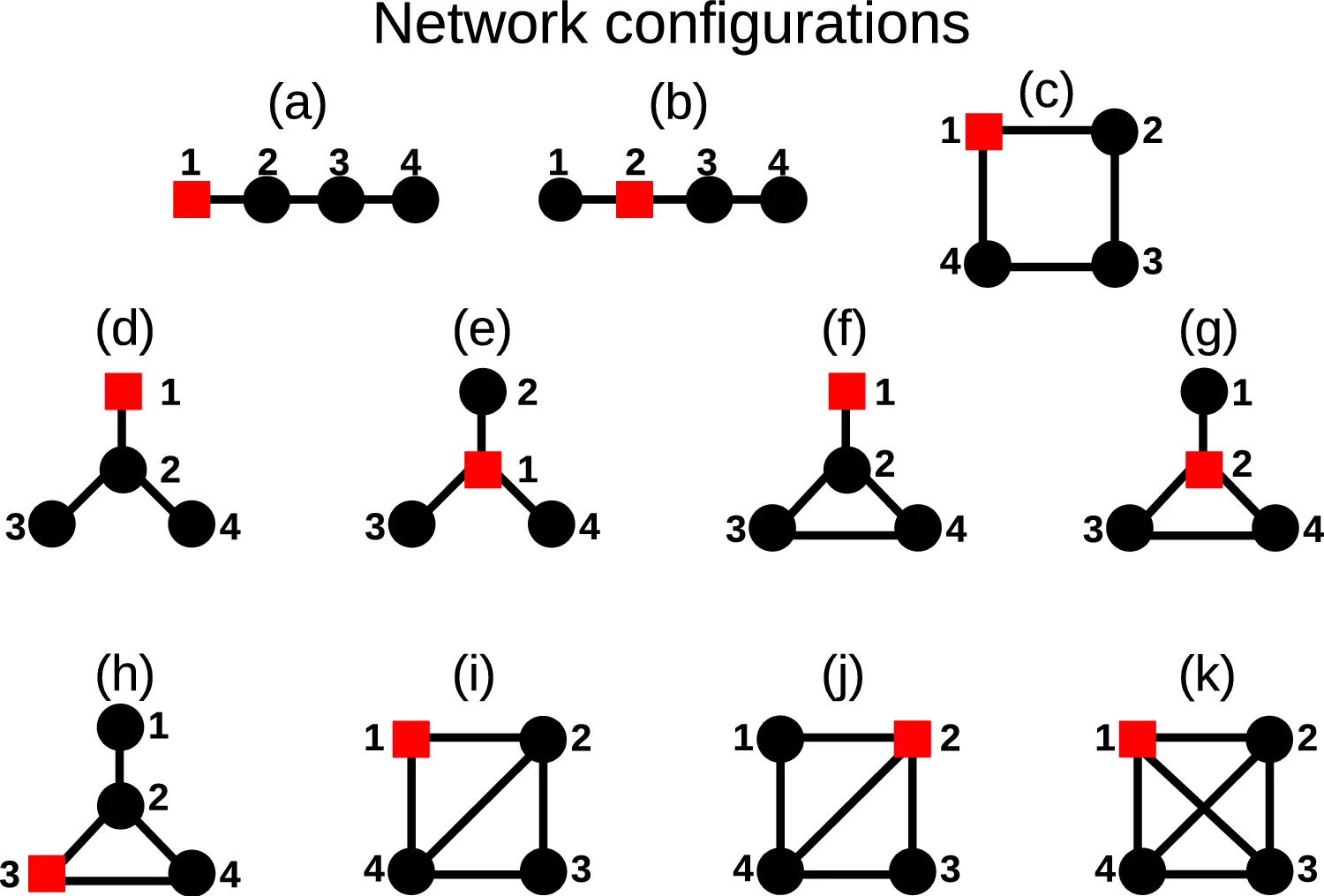
Network configurations considered in this study, each composed of four neurons. Black nodes denote bursting neurons, and the red node denotes the tonic neuron

We selected four neurons as a compromise between topological diversity and exhaustive analysis. There are six nonisomorphic connected simple graphs on four vertices: the path, star, cycle, paw, diamond, and complete graph. When one vertex is distinguished as the tonic neuron, placements related by graph automorphisms are equivalent. The numbers of inequivalent positions for these six graphs are 2, 2, 1, 3, 2, and 1, respectively, yielding 11 vertex-distinguished configurations. These configurations, shown in Fig. 2, therefore exhaust all connected four-node network topologies under the assumptions considered here and permit a complete motif-level comparison while keeping the neuronal composition and parameters fixed.

**Fig. 3:**
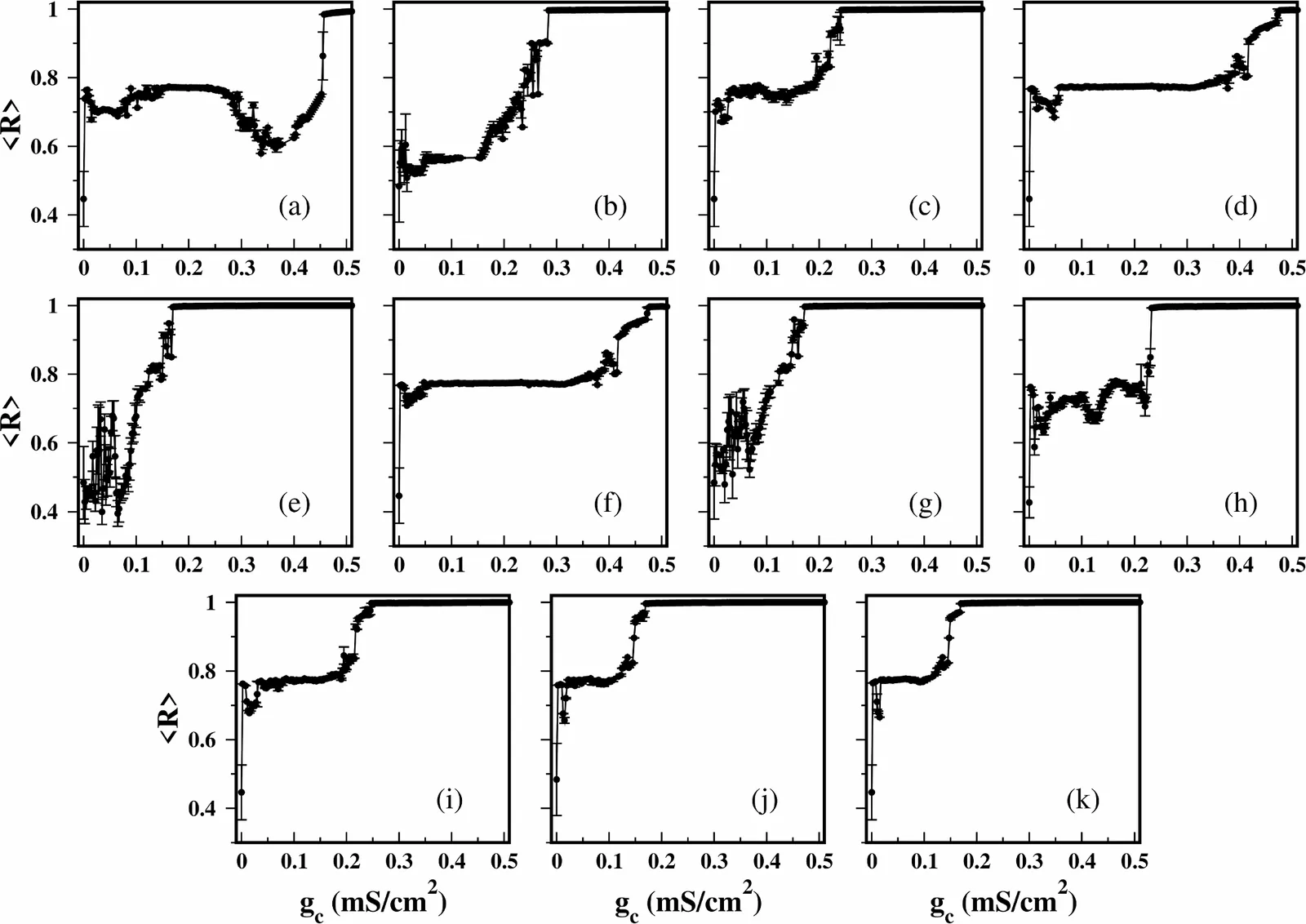
Time-averaged Kuramoto order parameter, $\langle R\rangle$, as a function of the electrical coupling strength $g_c$ for the 11 network configurations shown in Fig. 2. **a**–**k** follow the same configuration order as Fig. 2. At each coupling value, symbols denote the mean over ten forward-continuation realizations for different initial conditions, and error bars denote one sample standard deviation

To directly examine the development of global phase synchronization, Fig. 3 shows the time-averaged Kuramoto order parameter $\langle R\rangle$ as a function of the electrical coupling strength $g_c$ for the 11 network configurations. At each coupling value, the black circles represent the mean over ten forward-continuation realizations initialized from distinct initial conditions, while the error bars denote one sample standard deviation.

The curves show that both the route toward synchronization and the coupling strength at which it occurs depend on the network configuration. Configurations in which the tonic neuron occupies a more central position generally reach global synchronization at lower coupling strengths, although gradual, abrupt, and nonmonotonic transitions are observed. Although the variability increases in some transition regions, the principal topology-dependent trends remain consistent across the initial conditions considered. Moreover, the independent-point protocol produced qualitatively equivalent curves, with only minor quantitative differences near some transition regions, showing that the main conclusions are not artifacts of the forward-continuation procedure.

To investigate the role of the distinct node (the tonic neuron) in each network, we evaluated its topological importance using two centrality measures: degree centrality (*DC*) and quasi-Laplacian centrality ($L_Q$). The degree centrality of node $v_i$, used here in its unnormalized form, is defined as

15 $$\begin{aligned} DC(v_i)=\sum _{j=1}^{N}A_{ij}, \end{aligned}$$

where $A_{ij}=1$ if nodes $v_i$ and $v_j$ are connected and $A_{ij}=0$ otherwise, and *N* is the number of nodes. Since all the networks considered here have the same size, *N=4*, normalization by *N-1* would not affect the relative comparisons among configurations.

The $L_{Q}$ is based on the quasi-Laplacian energy of a network (Ma et al. 2019), defined as

16 $$\begin{aligned} E_{Q}(G)=\sum _{i=1}^{n}\gamma _{i}^{2}, \end{aligned}$$

where $\gamma _{i}$ are the eigenvalues of the quasi-Laplacian matrix, *Q*(*G*). This matrix is given by *Q(G)=A(G)+D(G)*, where *A*(*G*) is the adjacency matrix and *D*(*G*) is the degree matrix of the network *G*. For a node $v_{i}\in V(G)$, let $G_{i}=G-v_{i}$ be the graph obtained from *G* by deleting $v_{i}$ and its adjacent edges. The quasi-Laplacian centrality of $v_{i}$ is then defined as

17 $$\begin{aligned} L_{Q}(v_{i},G)=E_{Q}(G)-E_{Q}(G_{i}). \end{aligned}$$

Here, the additive form *Q=D+A* is adopted following the original definition of quasi-Laplacian centrality (Ma et al. 2019). Although *Q* and the standard Laplacian *L=D-A* generally have different spectral properties, they yield identical energy-based vertex centralities for simple undirected graphs (Qi et al. 2013; Ma et al. 2019). Consequently, using either matrix would lead to the same centrality values and conclusions in the present analysis.

**Fig. 4:**
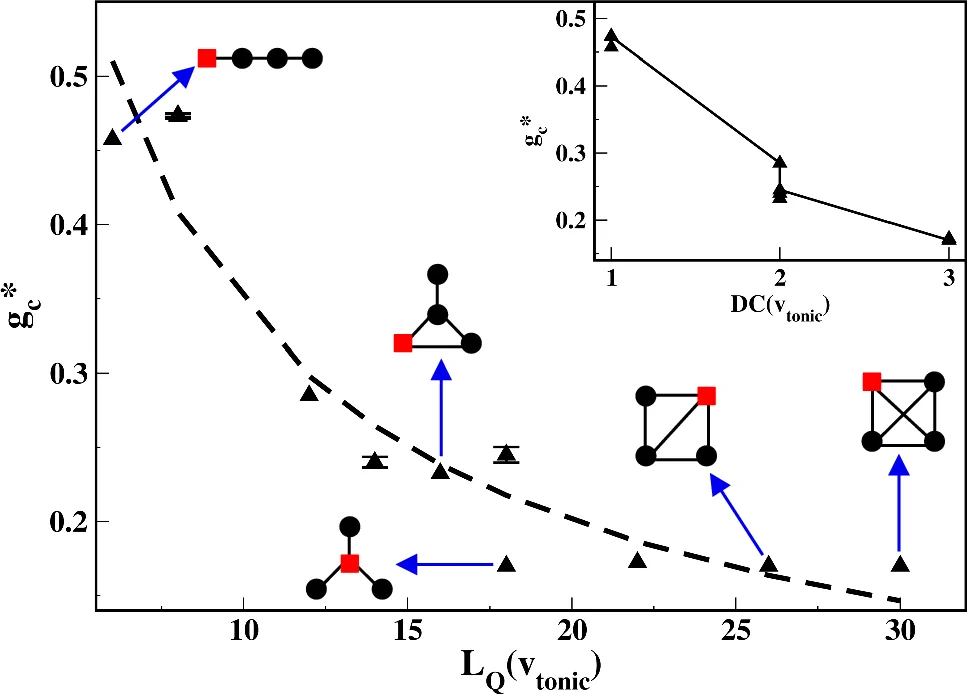
Critical coupling strength for global phase synchronization, $g_c^*$, as a function of the quasi-Laplacian centrality, $L_Q$, of the tonic neuron. Upward-pointing triangles represent the mean over ten forward-continuation realizations initialized from distinct initial conditions, and error bars denote one sample standard deviation. Error bars smaller than the symbols are not visible. The inset shows $g_c^*$ as a function of degree centrality, *DC*. Selected network configurations are illustrated, with the tonic neuron represented by a red square and the bursting neurons by black circles

The relationship between the critical coupling strength $g_{c}^{*}$ and these centrality measures is shown in Fig. 4.

A decreasing trend in $g_{c}^{*}$ is observed as both $L_Q$ and *DC* increase, indicating that networks in which the tonic neuron is more connected require weaker coupling to achieve synchronization. Using the mean values obtained from the ten realizations, the power-law fit (shown as a dashed curve) is

18 $$\begin{aligned} g_c^{*}=2.0473L_Q^{-0.7754}, \end{aligned}$$

with $R^{2}=0.903$. The 95% confidence intervals are [1.2165, 3.4457] for the prefactor and *[-0.9671,-0.5837]* for the exponent. The alternative exponential fit, $g_c^{*}=0.5745\exp (-0.04945L_Q)$, gives $R^{2}=0.808$, with corresponding intervals [0.4162, 0.7931] and *[-0.06761,-0.03129]*. Therefore, the power-law form provides the better empirical fit over the range considered.

Moreover, the critical coupling values cluster into three distinct groups depending on the degree centrality of the tonic neuron: networks with *DC=1* exhibit the highest $g_{c}^{*}$ values, followed by those with *DC=2*, and then those with *DC=3*, which show the lowest critical couplings.

### Effects on firing pattern transitions

To further investigate the impact of coupling strength on neural activity, we computed bifurcation diagrams of the interspike interval (ISI) from membrane potentials across different network configurations. This approach also enabled us to assess how network topology and tonic neuron position shape firing patterns.

**Fig. 5:**
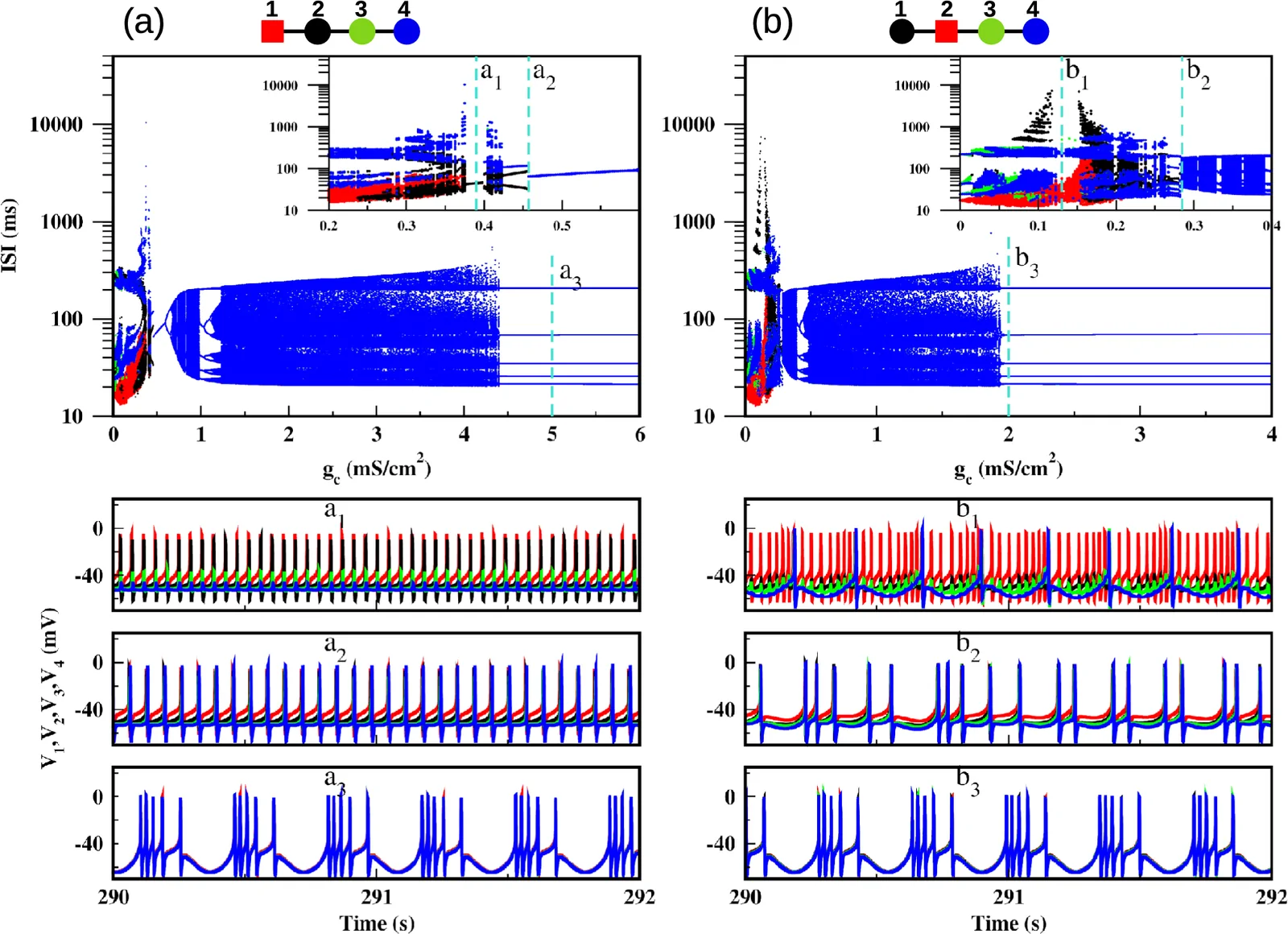
ISI bifurcation of each neuron as a function of the coupling strength for linear chain networks. In **a** the tonic neuron is placed at the first node, and in **b** at the second node, as shown in the network diagrams at the top of the figure. Time series are displayed for three values of $g_{c}$: (a1) $g_{c}=0.39$, (a2) $g_{c}=0.4575$, and (a3) $g_{c}=5$ for configuration (**a**); (b1) $g_{c}=0.13$, (b2) $g_{c}=0.285$, and (b3) $g_{c}=2$ for configuration (**b**). In both the time series and bifurcation diagrams, the tonic neuron is shown in red, while the bursting neurons are shown in black, green, and blue

The results for the linear chain network are shown in Fig. 5. As the coupling strength $g_{c}$ increases, distinct transitions in firing patterns emerge. In configuration (a), where the tonic neuron occupies position 1, the bursting neurons 3 and 4 (green and blue nodes) exhibit increasing ISIs, after which neurons 3 and 4 transition into subthreshold oscillations, while the tonic neuron and neuron 2 maintain period-1 spiking activity (Fig. 5a1). This coexistence occurs within the interval $g_{c} \in [0.3775, 0.3975]$. As coupling increases further, the neurons synchronize in tonic spiking, as shown in Fig. 5a2. Subsequently, the network exhibits a range of firing patterns (including tonic, periodic, and chaotic spiking, as well as periodic and chaotic bursting) before ultimately settling into periodic bursting(Fig. 5a3). In configuration (b), bursting neuron 1 exhibits increasing ISIs as the coupling strength increases, eventually transitioning to subthreshold oscillations. Within the interval $g_c \in [0.12, 0.15]$, the tonic neuron displays high-frequency spiking, while neurons 3 and 4 exhibit lower-frequency spiking relative to the tonic neuron (Fig. 5b1). As coupling increases further, the neurons synchronize into a chaotic spiking regime (Fig. 5b2), and ultimately settle into periodic bursting with five spikes per burst (Fig. 5b3).

**Fig. 6:**
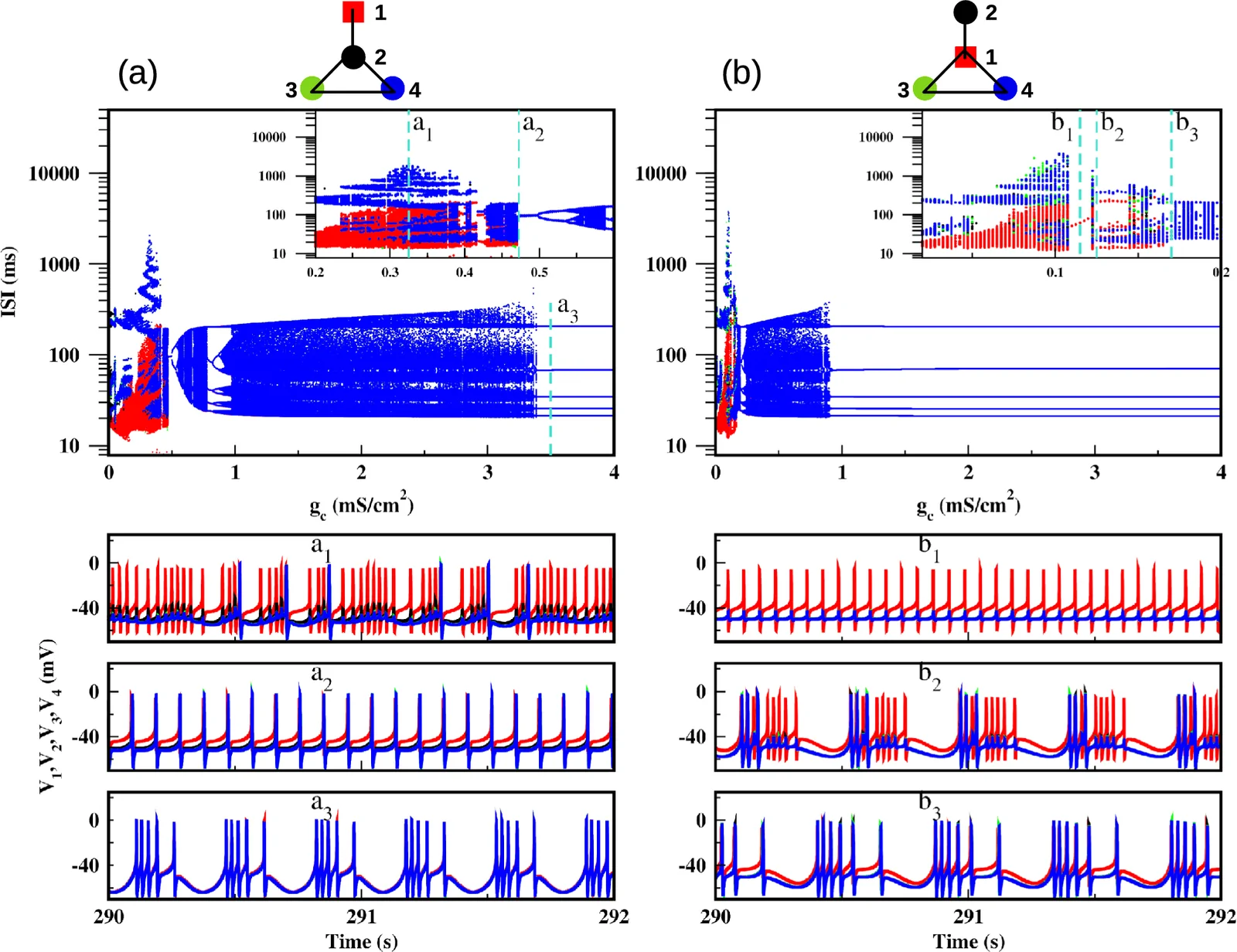
ISI bifurcation of each neuron as a function of the coupling strength for star networks. In **a** the tonic neuron is placed at the first node, and in **b** at the second node, as shown in the network diagrams at the top of the figure. Time series are displayed for three values of $g_{c}$: (a1) $g_{c}=0.325$, (a2) $g_{c}=0.4725$, and (a3) $g_{c}=3.5$ for configuration (**a**); (b1) $g_{c}=0.115$, (b2) $g_{c}=0.125$, and (b3) $g_{c}=0.17$ for configuration (**b**). In both the time series and bifurcation diagrams, the tonic neuron is shown in red, while the bursting neurons are shown in black, green, and blue

Figure 6 presents the bifurcation diagrams for a star network under two configurations. In configuration (a), the bursting neurons exhibit transitions characterized by large ISIs, forming a structure similar to the “stairway-to-heaven” pattern reported in Scully et al. (2025). These neurons synchronize among themselves, resulting in a state where three bursting neurons with high ISIs coexist with a tonic neuron firing at a higher frequency (Fig. 6a1). As coupling increases, the neurons first synchronize in a tonic spiking regime (Fig. 6a2) and eventually return to periodic bursting (Fig. 6a3). In configuration (b), bursting neurons (neurons 2, 3, and 4) exhibit increasing ISIs until they transition to subthreshold dynamics within the coupling interval $g_{c}\in [0.11,0.12]$, while the tonic neuron maintains tonic spiking, as depicted in Fig. 6b1. Afterward, bursting neurons synchronize among themselves, while the tonic neuron, now displaying bursting activity, does not synchronize with the others (Fig. 6b2). Because the peripheral neurons have no direct connections, this state suggests "remote synchronization". To distinguish this regime, we computed the direct and indirect phase-coherence measures introduced in the Sect. “Measuring synchronization”.

**Fig. 7:**
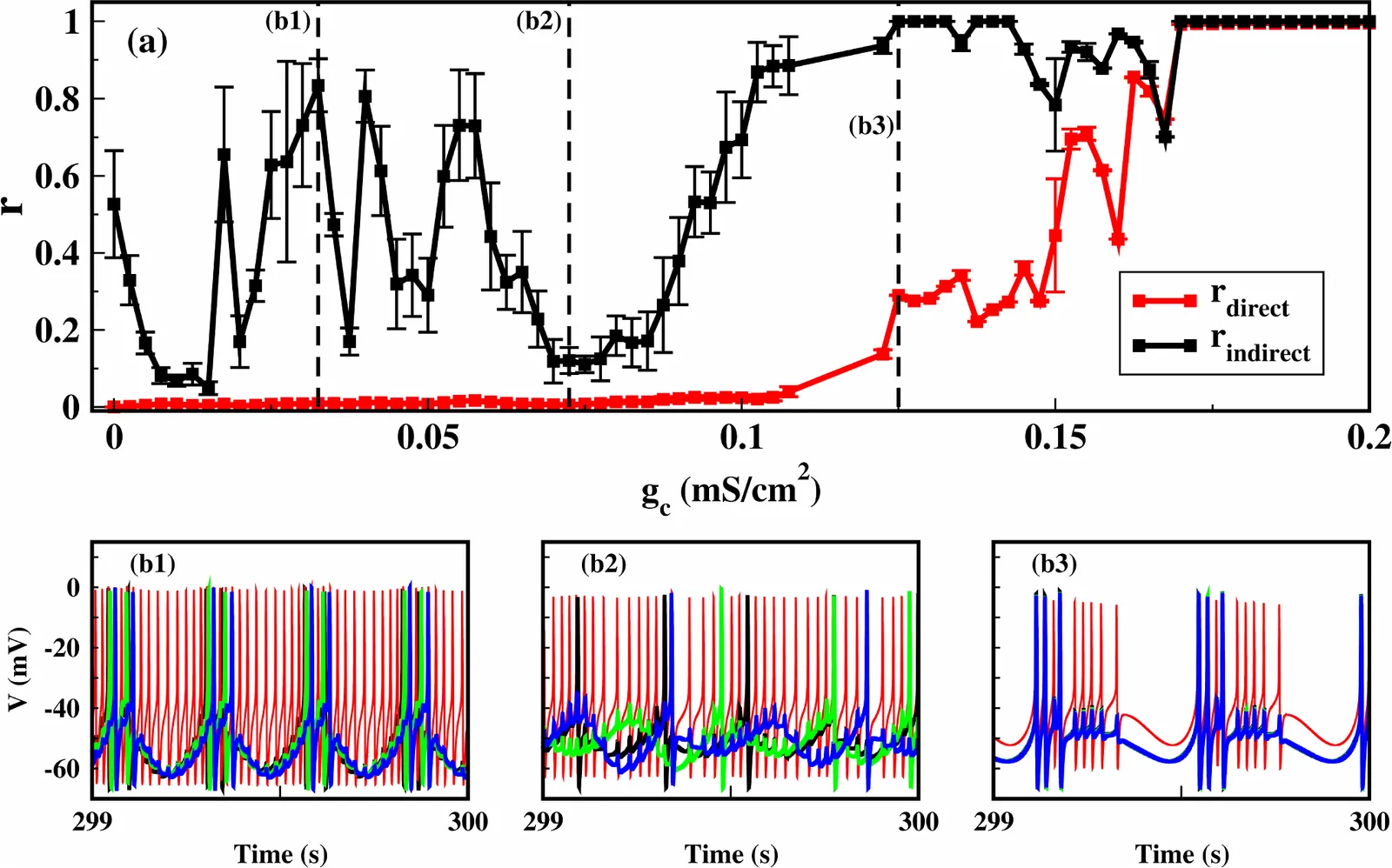
Quantification of remote synchronization in the star network with the tonic neuron located at the central node. **a** Mean direct coherence $r_{\textrm{direct}}$ between the central neuron and the three peripheral neurons (red) and mean indirect coherence $r_{\textrm{indirect}}$ among the three peripheral bursting neurons (black), as functions of the electrical coupling strength $g_c$. Symbols represent averages obtained from ten forward-continuation runs initialized from different initial conditions, and error bars denote the sample standard deviations. Vertical dashed lines indicate the coupling values selected for the representative voltage traces: (b1) $g_c=0.0325$, (b2) $g_c=0.0725$, and (b3) $g_c=0.125$. The central tonic neuron is shown in red, whereas the peripheral bursting neurons are shown in black, green, and blue

Figure 7 quantifies the phase-coherence structure of the star configuration in which the intrinsically tonic neuron occupies the hub. The peripheral bursting neurons are connected only through this central neuron. Remote synchronization is therefore identified when $r_{\textrm{indirect}}$ is high while $r_{\textrm{direct}}$ remains comparatively low. By contrast, low values of both quantities indicate the absence of a coherent cluster, whereas values close to unity for both quantities correspond to global phase coherence.

The dependence on $g_c$ is markedly nonmonotonic. At $g_c =0.0325$, the peripheral neurons exhibit appreciable mutual coherence while their coherence with the hub remains close to zero (Fig. 7b1). The relatively large error bars in this region indicate sensitivity to the initial condition. At $g_c=0.0725$, both coherence measures are low and the peripheral cluster is lost (Fig. 7b2). The clearest remote-synchronization regime occurs, for example at $g_c=0.125$, where $r_{\textrm{indirect}}$ is close to unity and $r_{\textrm{direct}}$ remains substantially lower (Fig. 7b3). The time series confirm that the three indirectly connected bursting neurons evolve coherently, while the central tonic neuron remains outside their synchronized cluster. At stronger coupling, both coherence measures approach unity, indicating the transition from remote synchronization to global phase coherence. Simulations performed without forward continuation produced qualitatively similar behavior, supporting the robustness of this sequence of regimes. The phenomenon is consistent with the structural scenario described by Bergner et al. (2012), although here the required dynamical mismatch arises from the intrinsic tonic-spiking and bursting regimes of conductance-based neurons coupled through electrical synapses.

**Fig. 8:**
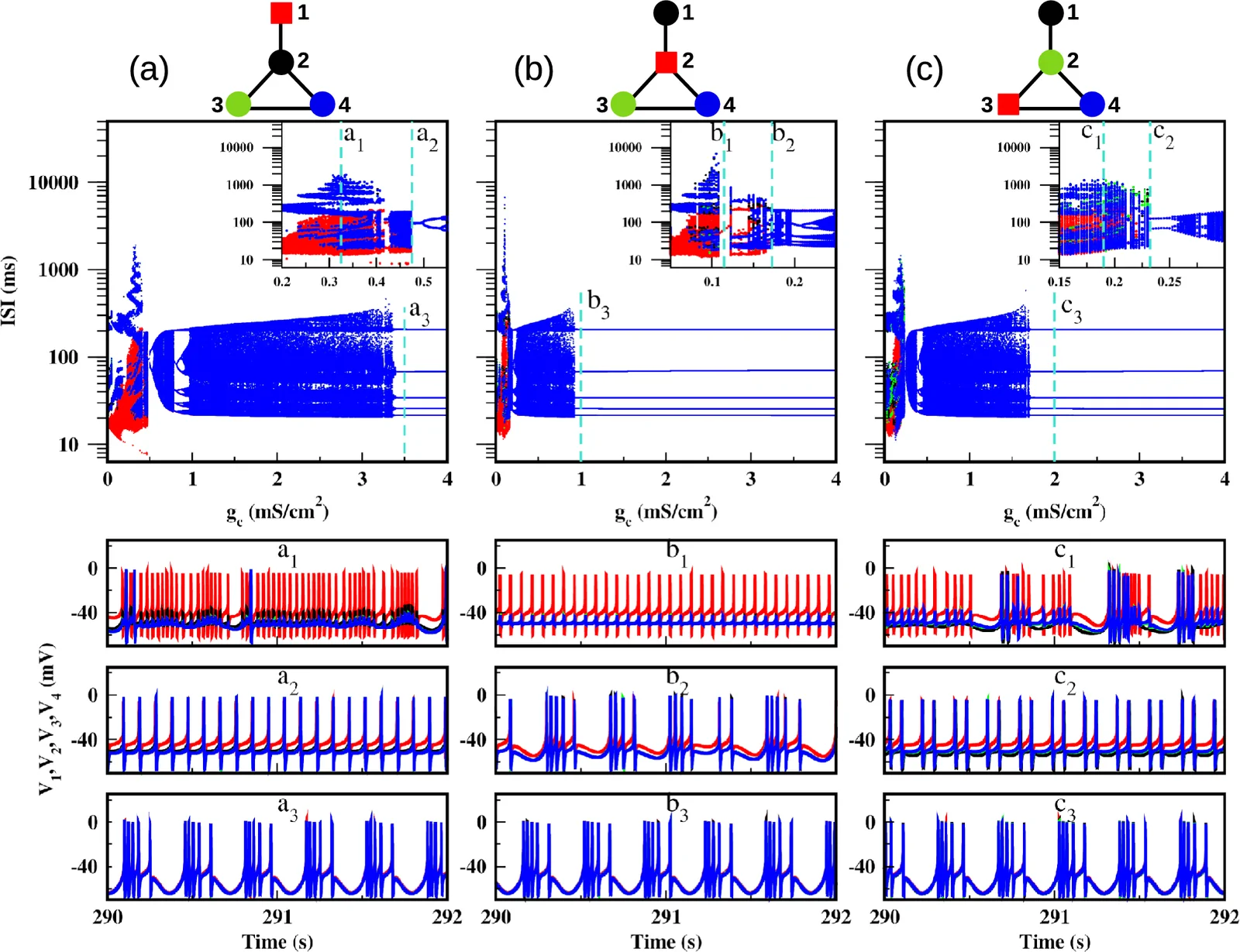
ISI bifurcation of each neuron as a function of the coupling strength for star-type networks. In **a** the tonic neuron is placed at the first node, and in **b** at the second node, as shown in the network diagrams at the top of the figure. Time series are displayed for three values of $g_{c}$: (a1) $g_{c}=0.325$, (a2) $g_{c}=0.475$, and (a3) $g_{c}=3.5$ for configuration (**a**); (b1) $g_{c}=0.115$, (b2) $g_{c}=0.1725$, and (b3) $g_{c}=1$ for configuration (**b**); (c1) $g_{c}=0.19$, (c2) $g_{c}=0.2325$, and (c3) $g_{c}=2$ for the configuration (**c**). In both the time series and bifurcation diagrams, the tonic neuron is shown in red, while the bursting neurons are shown in black, green, and blue

Figure 8 presents the bifurcation diagrams for a star-type network under three configurations. In configuration (a), where the tonic neuron is located at node 1, neurons 2, 3, and 4 rapidly synchronize, forming a cluster. Around $g_{c}=0.325$, their ISI values increase, while the tonic neuron maintains spiking dynamics, as shown in Fig. 8a1. As coupling increases, all neurons synchronize into tonic spiking (Fig. 8a2), followed by transitions through various firing patterns including periodic and chaotic spiking and both periodic and chaotic bursting before ultimately returning to periodic bursting with five spikes per burst (Fig. 8a3). In configuration (b), where the tonic neuron is located at node 2, the bursting neurons 1, 3, and 4 exhibit increased ISI values and transition to subthreshold oscillations within the coupling interval $g_{c} \in [0.11,0.12]$, as shown in Fig. 8b1. The neurons first synchronize into an irregular bursting regime (Fig. 8b2) and eventually transition to periodic bursting dynamics (Fig. 8b3). In configuration (c), where the tonic neuron is located at node 3, neurons 1, 2, and 4 exhibit a considerable increase in their ISIs (Fig. 8c1). Subsequently, the neurons synchronize into a period-2 spiking regime, as shown in Fig. 8c2 and then undergo several transitions before reaching a state of periodic bursting (Fig. 8c3).

**Fig. 9:**
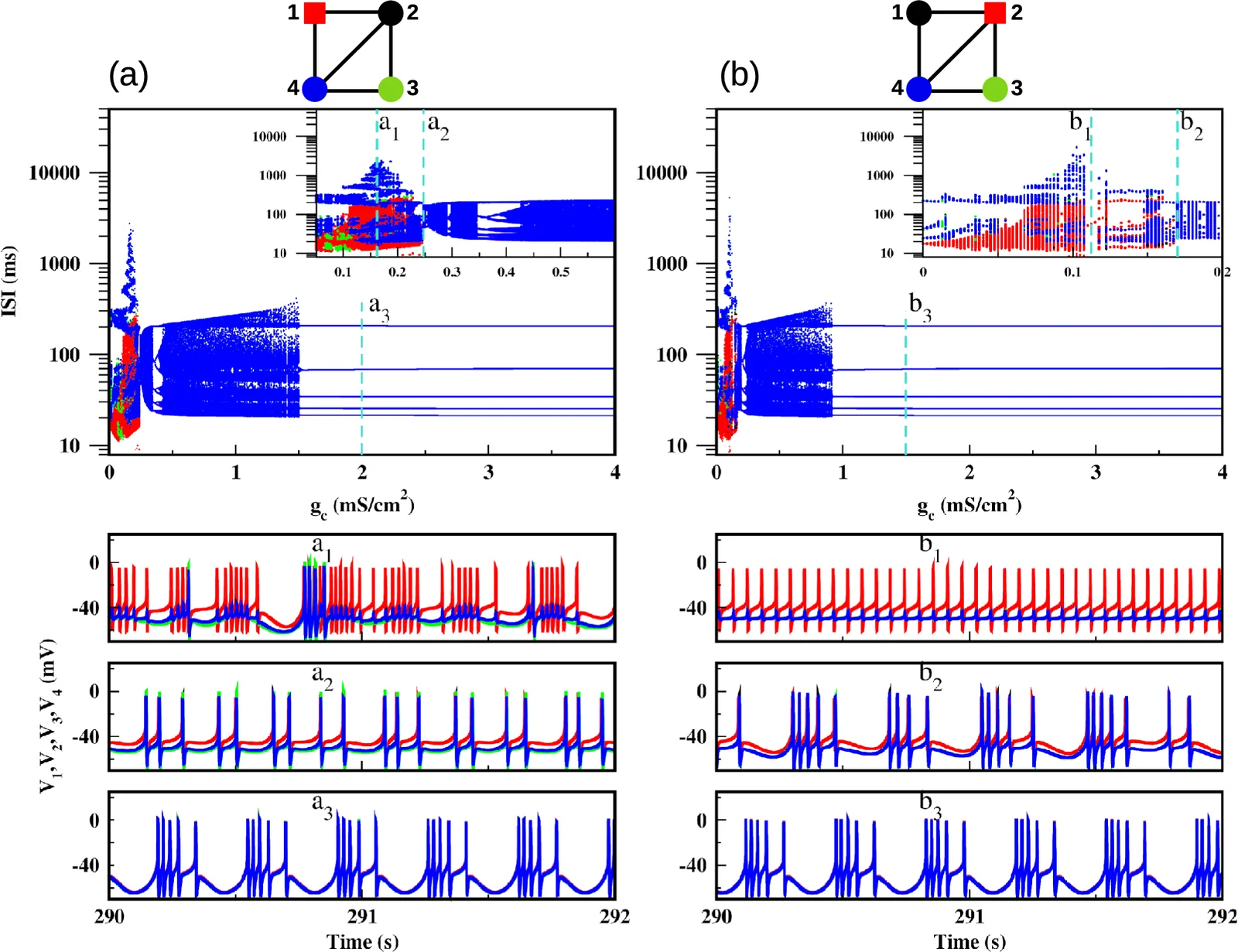
ISI bifurcation of each neuron as a function of the coupling strength for a square-type network. In **a** the tonic neuron is placed at the first node, and in **b** at the second node, as shown in the network diagrams at the top of the figure. Time series are displayed for three values of $g_{c}$: (a1) $g_{c}=0.1625$, (a2) $g_{c}=0.2475$, and (a3) $g_{c}=5$ for configuration (**a**); (b1) $g_{c}=0.1125$, (b2) $g_{c}=0.17$, and (b3) $g_{c}=1.5$ for configuration (**b**). In both the time series and bifurcation diagrams, the tonic neuron is shown in red, while the bursting neurons are shown in black, green, and blue

For the square-type network, the bifurcation diagrams are shown in Fig. 9. When the tonic neuron is located at node 1, bursting neurons 2, 3, and 4 synchronize among themselves, exhibiting increasing ISI values and forming a “stairway-to-heaven" structure around $g_{c}=0.1625$, while the tonic neuron remains desynchronized. As coupling increases, all neurons synchronize into a chaotic spiking regime (Fig. 9a2), followed by a sequence of transitions that culminate in periodic bursting dynamics (Fig. 9a3). In configuration (b), before synchronization occurs, the bursting neurons (neurons 1, 3, and 4) transition to subthreshold oscillations, as shown in Fig. 9b1. With further increases in coupling strength, the neurons synchronize into chaotic bursting dynamics (Fig. 9b2) and eventually return to periodic bursting (Fig. 9b3).

Finally, the firing pattern transitions observed in the ring and complete networks are similar to those described in the previous bifurcation diagrams. The corresponding bifurcation diagrams are provided in Appendix A.

**Fig. 10:**
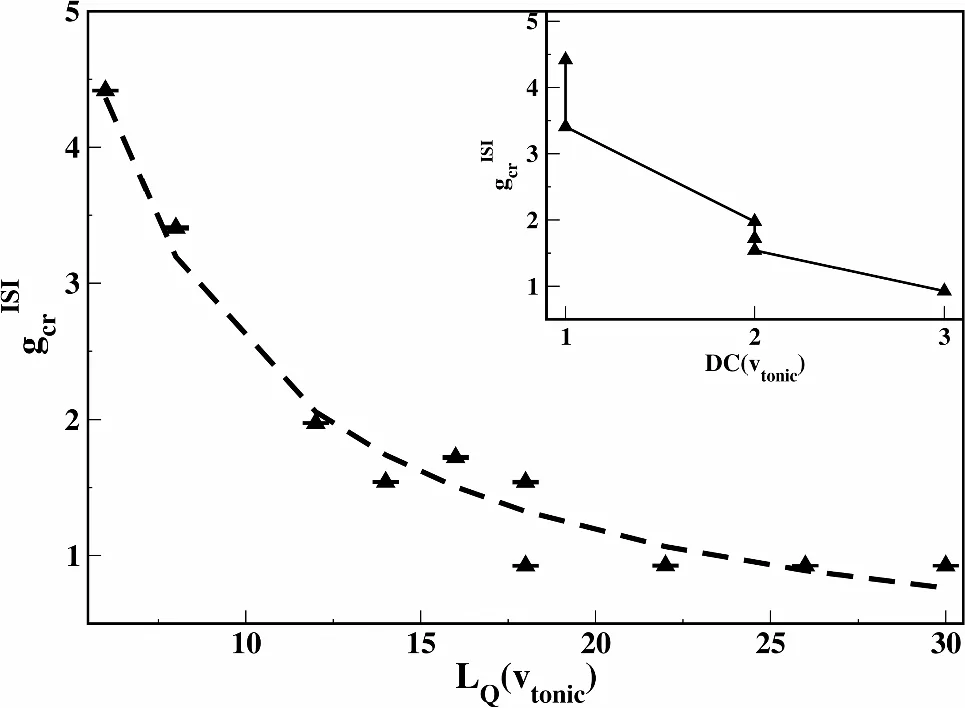
Critical coupling strength for the return to synchronized periodic bursting, $g_{cr}^{\textrm{ISI}}$, as a function of the quasi-Laplacian centrality, $L_Q$, of the tonic neuron. Upward-pointing triangles represent the mean over ten forward-continuation realizations initialized from distinct initial conditions, and error bars denote one sample standard deviation. Error bars smaller than the symbols are not visible. The inset shows $g_{cr}^{\textrm{ISI}}$ as a function of degree centrality, *DC*

As observed in the previous bifurcation diagrams, all network configurations eventually return to periodic bursting (five spikes per burst) for sufficiently strong coupling. However, the coupling strength at which this transition occurs depends on network topology. To quantify this behavior, we defined the critical coupling value $g_{cr}^{ISI}$ as the point in the ISI bifurcation diagrams where the distribution of ISIs changes from a dense scatter (irregular bursting) to five well-defined clusters, corresponding to a stable 5-spikes-per-burst periodic regime. We then analyzed the relationship between $g_{cr}^{ISI}$ and the centrality measures (quasi-Laplacian centrality and degree); the results are presented in Fig. 10. The data points are empirically described by a power-law fit (dashed curve), given by

19 $$\begin{aligned} g_{cr}^{\textrm{ISI}}=30.503L_Q^{-1.0852}, \end{aligned}$$

with $R^{2}=0.925$. The 95% confidence intervals are [16.224, 57.351] for the prefactor and *[-1.3176,-0.8527]* for the exponent. The exponential alternative, $g_{cr}^{\textrm{ISI}}=5.1582\exp (-0.06927L_Q)$, gives $R^{2}=0.830$, with corresponding intervals [3.3904, 7.8480] and *[-0.09291,-0.04564]*. Thus, the power-law form also provides the better empirical fit for this transition.

In contrast to the behavior observed for $g_{c}^{*}$, degree centrality alone is insufficient to fully characterize the dependence of $g_{cr}^{ISI}$ on the network structure.

**Table 1:** Summary of the synchronization dynamics for the 11 network configurations. The critical coupling is reported as the mean ± standard deviation over ten realizations with different initial conditions. The qualitative classifications were consistent across all realizations. Values of$g_c$are given in$\mathrm {mS/cm^2}$.

Configuration (Fig. 2)	$L_Q$	*DC*	$g_c^{*}\pm \sigma _{g_c^*}$	Synchronization dynamics at $g_c^*$	Partial firing death
(a)	6	1	*0.4575± 0.0000*	Tonic spiking	Yes
(d)	8	1	*0.473± 0.00158*	Tonic spiking	No
(f)	8	1	*0.47375± 0.00132*	Tonic spiking	No
(b)	12	2	*0.285± 0.0000*	Chaotic spiking	Yes
(c)	14	2	*0.24± 0.00354*	Chaotic spiking	No
(h)	16	2	*0.2325± 0.0000*	Period-2 spiking	No
(i)	18	2	*0.245± 0.00527*	Chaotic spiking	No
(e)	18	3	*0.17± 0.0000*	Period-6 bursting	Yes
(g)	22	3	*0.1725± 0.0000*	Chaotic bursting	Yes
(j)	26	3	*0.17± 0.0000*	Chaotic bursting	Yes
(k)	30	3	*0.17± 0.0000*	Chaotic bursting	Yes

Table 1 summarizes the synchronization regimes observed at the critical coupling strength for all network topologies considered, along with the $L_{Q}$ and *DC* values of the tonic neuron. We find that networks in which the tonic neuron has low $L_{Q}$ and *DC=1* first synchronize in tonic spiking. As coupling increases further, synchronous tonic-to-bursting transitions then occur. Networks with intermediate $L_{Q}$ and *DC=2* first synchronize in spiking regimes, whereas those with high $L_{Q}$ and *DC=3* initially exhibit synchronized bursting dynamics.

The two configurations with $L_Q=18$ illustrate the limitations of using a single scalar centrality measure. In the square-type configuration of Fig. 2i, the tonic neuron has *DC=2*, and the network synchronizes at $g_c^{*}=0.245\pm 0.00527$ into chaotic spiking, with $g_{cr}^{ISI}=1.5405\pm 0.0073$ and no partial firing death. By contrast, in the hub-centered star configuration of Fig. 2e, the tonic neuron has *DC=3*, and the network synchronizes at $g_c^{*}=0.1700$ into period-6 bursting, with $g_{cr}^{ISI}=0.9255\pm 0.0047$ and partial firing death. Therefore, although $L_Q$ incorporates both the degree of the tonic neuron and the connectivity of its neighbors, it is not a unique predictor of the dynamics. Degree centrality distinguishes this pair, showing that $L_Q$ and *DC* should be interpreted as complementary structural descriptors.

These findings highlight the importance of the network configuration in determining the initial synchronized regime; that is, with neuron parameters fixed, simply changing the network structure and the position of the tonic neuron can alter the resulting synchronization dynamics. Furthermore, the coexistence of neurons with spiking dynamics and subthreshold oscillations (partial firing death) also depends on the network configuration and tonic neuron position, as shown in Table 1. In particular, we observed that in networks where the tonic neuron has *DC=3*, there was always a coupling interval exhibiting this behavior.

## Discussion and conclusion

In this study, we investigated synchronization dynamics in small neuronal networks composed of one tonic neuron and three bursting neurons coupled via electrical synapses. We found that both the critical coupling for global phase synchronization and the firing-pattern transitions depend sensitively on the tonic neuron’s topological role. In particular, tonic neurons with higher quasi-Laplacian centrality or degree centrality were associated with markedly lower critical couplings.

The reported power-law expressions should be interpreted only as empirical fits over the eleven motifs considered here, rather than as universal scaling laws. Because quasi-Laplacian centrality incorporates both the degree of the tonic neuron and the connectivity of its neighbors (Ma et al. 2019), it can serve as a proxy for local structural influence. Since diffusive electrical coupling depends on the product of $g_c$ and topology-dependent voltage differences, an inverse relation between structural influence and the coupling required to reach the corresponding collective transition is heuristically plausible. This reasoning is consistent with the decreasing trend but does not analytically derive the exponents. Furthermore, the fitted exponents may depend on the neuronal model, intrinsic parameters, tonic–bursting mismatch, motif ensemble, and coupling mechanism. Standard spectral approaches based on the coupling Laplacian primarily characterize the stability of synchronous states in networks of identical oscillators (Pecora and Carroll 1998); consequently, they do not by themselves directly predict these empirical fits, which involve heterogeneous dynamics and firing-pattern transitions.

Our analysis of ISI bifurcation diagrams revealed complex firing-pattern transitions as coupling strength increased. These included sequences of subthreshold oscillations, tonic spiking, periodic and chaotic spiking, and both chaotic and periodic bursting. Several network configurations exhibited large-ISI “stairway-to-heaven” structures. Similar ISI sequences have previously been associated with trajectories approaching a homoclinic orbit (Scully et al. 2025). However, the present evidence is limited to the ISI diagrams and does not establish a homoclinic mechanism; confirming this interpretation would require dedicated phase-space, return-map, and bifurcation analyses. We also observed remote synchronization in the star configuration, where the peripheral bursting neurons synchronized despite being connected only indirectly through the tonic hub, which remained outside their synchronized cluster. This behavior is consistent with a relay mechanism: the star topology and diffusive electrical coupling allow the peripheral neurons to interact through the hub, while the intrinsic tonic–bursting mismatch provides a plausible explanation for the lower coherence of the hub with the peripheral cluster over an intermediate coupling range (Bergner et al. 2012). A detailed stability analysis would nevertheless be required to establish the precise mechanism in the Huber–Braun model.

Previous studies with the Huber–Braun model [e.g., Shaffer et al. (2016, 2017); Follmann et al. (2018)] have highlighted tonic-to-bursting transitions as functions of intrinsic dynamics and coupling strength. Our results extend these findings by showing that changes in network topology and the tonic neuron’s position alone can drive such transitions. Furthermore, previous observations of spike-suppressed or subthreshold states arising from interactions among heterogeneous Huber–Braun neurons—where heterogeneity was introduced through temperature variability (Tchaptchet 2018)—are consistent with our results, in which heterogeneity is instead controlled through the slow-repolarizing conductance $g_{sr}$ and gives rise to partial firing death, that is, the coexistence of neurons exhibiting subthreshold oscillations and spiking. Firing death refers to the suppression of spiking activity and the emergence of subthreshold oscillations caused by mutual interactions (Ciszak et al. 2013; Hennig and Schimansky-Geier 2007).

In our networks, partial firing death was observed only in bursting neurons, but it was not restricted to a particular degree of the tonic neuron. It occurred in the two linear-chain configurations, in which the tonic neuron has degree one or two, and was also observed in the configurations where it has degree three. Thus, the tonic-neuron degree alone does not determine its emergence. Its occurrence across different motifs suggests that partial firing death results from the combined influence of tonic–bursting heterogeneity and the topology-dependent distribution of diffusive inputs. Specifically, the connection pattern determines which bursting neurons receive direct input from the tonic neuron, which are influenced only indirectly through other bursting neurons, and how the bursting neurons interact with each other. Because the net coupling current received by each neuron depends on the voltage differences with its connected neighbors, these different local arrangements may suppress spiking in selected bursting neurons while the remaining neurons stay active. Identifying the underlying bifurcation mechanism, however, requires a dedicated analysis beyond the scope of the present study.

The present findings emphasize the importance of single-neuron position for synchronization dynamics in small neuronal assemblies and may have biological relevance. Abnormally high levels of synchronization are associated with several brain pathologies (Uhlhaas and Singer 2006). In particular, interactions between tonic and bursting firing patterns play a key role in seizure dynamics (Fröhlich et al. 2008; Timofeev et al. 1998; Sorokin et al. 2017). Consistent with this, Follmann et al. (2024) used the Huber–Braun model to demonstrate wave-like synchronization reminiscent of focal seizures, in which a centrally located tonic neuron more effectively entrains surrounding bursting neurons at elevated temperatures, supporting the view of tonic neurons as pacemakers of pathological rhythms. Moreover, the coexistence of neurons exhibiting high-frequency spiking with others displaying very low firing rates or partial firing death in our motifs closely resembles heterogeneous single-unit activity observed in the human neocortex during epileptic seizures (Truccolo et al. 2011). Our results demonstrate that similar coexistence patterns can arise naturally in minimal heterogeneous networks as a consequence of coupling strength, topology, and the positional role of a single tonic neuron. This suggests that complex ictal-like heterogeneity does not necessarily require large-scale networks but may already be encoded at the level of small neuronal motifs.

While our analysis provides theoretical insight into how topology and coupling govern synchronization in heterogeneous neuronal motifs, its scope is restricted to bidirectional electrical coupling. The diffusive gap-junction coupling considered here generates a current proportional to the voltage difference between connected neurons and therefore tends to reduce that difference. By contrast, chemical synapses are typically directed and nonlinear, with their effects determined by reversal potentials, synaptic kinetics, and transmission delays. Previous modeling studies have shown that electrical and inhibitory coupling can stabilize different phase relationships depending on coupling strength and synaptic kinetics (Lewis and Rinzel 2003), that transmission delays can modify the coupling required for synchronization in networks of bursting neurons (Dhamala et al. 2004), and that electrical, chemical, and mixed coupling can produce distinct synchronized spiking and bursting patterns (Çakir 2016). Accordingly, incorporating excitatory or inhibitory chemical synapses, or mixed electrical–chemical coupling, could shift the critical coupling thresholds, alter the phase relationships and firing-pattern transitions, and modify the relative influence of different tonic-neuron positions. Thus, whether the positional effects, quantitative relations, and dynamical regimes reported here persist under chemical or mixed coupling remains an open question requiring separate systematic investigation.

The small spread in $g_{sr}$ among the three bursting neurons was introduced intentionally to represent weak intrinsic variability. To assess whether this choice materially affected the results, we performed additional simulations with identical bursting neurons, as well as a limited set of realizations with small independently sampled variations around the original $g_{sr}$ values for the tonic neuron and each bursting neuron. Although these checks are not presented here as separate figures, the tested cases qualitatively preserved the main topology-dependent synchronization and firing-pattern trends, indicating that the small conductance spread does not materially affect the main conclusions within the local parameter regime studied. However, these limited tests do not establish robustness over the full parameter space of the Huber–Braun model. A systematic exploration of $g_{sr}$, temperature, and the effects of noise is left for future work.

Backward-continuation tests for selected configurations revealed minor forward–backward differences in some firing-pattern transitions, suggestive of narrow hysteretic intervals, without altering the main topology-dependent trends. A systematic analysis of hysteresis and multistability would require mapping coexisting attractors and their basins of attraction across all network configurations and would constitute a separate level of analysis beyond the objectives of the present study. Such an investigation represents an interesting direction for future work.

In conclusion, our results demonstrate that network topology—and, in particular, the topological role of a single tonic neuron—is a key factor governing the emergence of collective rhythms in electrically coupled neuronal networks. A network-motif perspective therefore provides a valuable framework for linking structure to function in neuronal systems (Fan et al. 2024; Curto and Morrison 2019), helping to elucidate how complex collective dynamics can arise from minimal network motifs under both physiological and pathological conditions.

## Figures and Tables

**Fig. 11:**
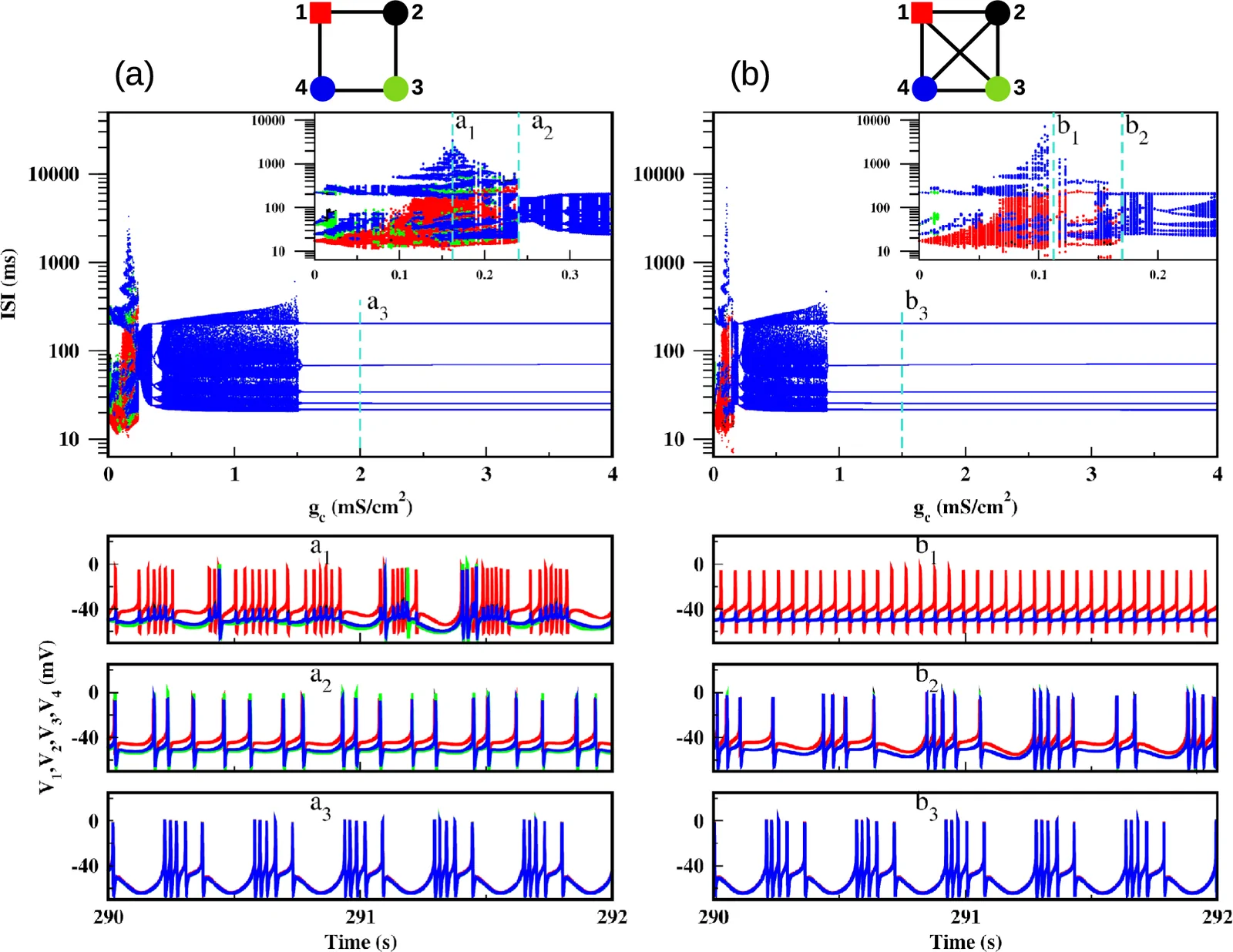
ISI bifurcation of each neuron as a function of the coupling strength for regular networks. In **a** the tonic neuron is placed at the first node, and in **b** at the second node, as shown in the network diagrams at the top of the figure. Time series are displayed for three values of $g_{c}$: (a1) $g_{c}=0.1625$, (a2) $g_{c}=0.24$, and (a3) $g_{c}=5$ for the configuration (**a**); (b1) $g_{c}=0.1125$, (b2) $g_{c}=0.17$, and (b3) $g_{c}=2$ for the configuration (**b**). In both the time series and bifurcation diagrams, the tonic neuron is shown in red, while the bursting neurons are shown in black, green, and blue

## Data Availability

No datasets were generated or analysed during the current study.

## Appendix A Ring and complete network

The bifurcation diagrams for the ring and complete configurationsare shown in Fig. 11.
